# High resolution melting analysis for a rapid identification of heterozygous and homozygous sequence changes in the *MUTYH *gene

**DOI:** 10.1186/1471-2407-11-305

**Published:** 2011-07-21

**Authors:** Rossella Tricarico, Francesca Crucianelli, Antonio Alvau, Claudio Orlando, Roberta Sestini, Francesco Tonelli, Rosa Valanzano, Maurizio Genuardi

**Affiliations:** 1 Department of Clinical Pathophysiology, Medical Genetics Unit, University of Florence, Florence, Italy; 2 Department of Surgery, University of Siena, Siena, Italy; 3 Current Address: Department of Veterinary and Animal Sciences, University of Massachusetts, Amherst, USA; 4 Department of Clinical Pathophysiology, Clinical Biochemistry Unit, University of Florence, Florence, Italy; 5 Department of Clinical Pathophysiology, Surgery Unit, University of Florence, Florence, Italy; 6 Fiorgen Foudation for Pharmacogenomics, Sesto Fiorentino, Italy

**Keywords:** HRMA, *MUTYH*, colorectal cancer, polyposis, mutation

## Abstract

**Background:**

*MUTYH*-associated polyposis (MAP) is an autosomal recessive form of intestinal polyposis predisposing to colorectal carcinoma. High resolution melting analysis (HRMA) is a mutation scanning method that allows detection of heterozygous sequence changes with high sensitivity, whereas homozygosity for a nucleotide change may not lead to significant curve shape or melting temperature changes compared to homozygous wild-type samples. Therefore, HRMA has been mainly applied to the detection of mutations associated with autosomal dominant or X-linked disorders, while applications to autosomal recessive conditions are less common.

**Methods:**

*MUTYH *coding sequence and UTRs were analyzed by both HRMA and sequencing on 88 leukocyte genomic DNA samples. Twenty-six samples were also examined by SSCP. Experiments were performed both with and without mixing the test samples with wild-type DNA.

**Results:**

The results show that all *MUTYH *sequence variations, including G > C and A > T homozygous changes, can be reliably identified by HRMA when a condition of artificial heterozygosity is created by mixing test and reference DNA. HRMA had a sensitivity comparable to sequencing and higher than SSCP.

**Conclusions:**

The availability of a rapid and inexpensive method for the identification of *MUTYH *sequence variants is relevant for the diagnosis of colorectal cancer susceptibility, since the MAP phenotype is highly variable.

## Background

Colorectal carcinoma (CRC) is the second most common cause of cancer-related mortality in developed countries. About 1% of CRCs arise in individuals affected with familial adenomatous polyposis. This condition can be caused by mutations in at least 2 distinct genes: *APC*, implicated in the autosomal dominant form, and *MUTYH*, which is involved in *MUTYH*-associated polyposis (MAP: MIM#608456). MAP is transmitted as an autosomal recessive trait due to biallelic mutations of the *MUTYH *gene, whose product is a DNA glycosylase that removes adenine from A•8-oxoG as part of the base excision repair process [1].

MAP patients are homozygotes or compound heterozygotes for mutations of the *MUTYH *gene, that is comprised of 16 exons. Two missense base substitutions, c.536A > G (p.Tyr179Cys) and c.1187G > A (p.Gly396Asp), located in exons 7 and 13, respectively, account for about 75% of pathogenic *MUTYH *allelic variants reported among Caucasians [2]. In addition, other recurrent *MUTYH *mutations have been identified in Pakistani, Dutch, Portuguese and Japanese patients [3,4]. The remaining fraction of *MUTYH *variants identified in MAP patients is highly heterogeneous and can be located along the whole coding sequence.

The identification of *MUTYH *germline mutations has clinical relevance because the MAP phenotype is variable and often difficult to identify on clinical grounds alone [5-7]. The first step in *MUTYH *molecular screening in Caucasian patients is usually represented by the search for the two common mutations. Complete analysis of the *MUTYH *coding region is then carried out when none or only one mutant allele is identified with the first screening.

Several scanning techniques are currently performed to identify *MUTYH *sequence variants. These are usually based on direct sequencing, often preceded by denaturating high-performance liquid chromatography (dHPLC), single strand conformational polymorphism (SSCP) or Tetra-ARMS-PCR assay [8-10]. Despite their high degree of accuracy, these methods are relatively time-consuming and expensive.

High resolution melting analysis (HRMA) is a mutation detection method based on the principle that the melting curves of DNA fragments vary depending on base composition, and is a potentially useful technique for fast genotyping and high-throughput mutation scanning in genetic diagnosis [11,12]. Heterozygous samples are identified by differences in melting curve shape. When a nucleotide change is present in a target sequence, the resulting melting curve of the PCR product is a composite of both heteroduplex and homoduplex components that is visualized by the different melting curve shapes of the two species present. Homozygous base changes are not associated with heteroduplex formation. Since DNA sequences differing in a single base often have different melting temperatures (T_m_), discrimination between homozygous wild-type and homozygous mutant samples can be achieved by comparing the T_m _values of test samples with those of wild-type controls.

Unlike other scanning methods, mutation analysis by HRMA provides a closed-tube system that reduces the risk of contamination, decreases analytical time and requires no sample processing or separation after PCR. HRMA has been used for the detection of mutations associated with human genetic diseases, especially autosomal dominant or X-linked disorders [13-15]. More recently, this method was used to identify sequence variations in autosomal recessive disorders, such as primary carnitine deficiency, cystic fibrosis, and Zellweger syndrome [16-19]. This technique has also been used to identify specific common variants in selected *MUTYH *exons [20,21]. However, there is a lack of information on the sensitivity of HRMA for the identification of a broader spectrum of *MUTYH *mutations.

In this study, we report on an HRMA-based approach for the rapid detection of *MUTYH *variations spread along the entire coding region, as well as in the 5' and 3' untranslated regions, in both the heterozygous and homozygous state.

## Methods

### DNA samples

The study was carried out on 88 genomic DNA samples from unrelated individuals. We initially analyzed a set of 26 samples of known *MUTYH *genotype, previously investigated by both SSCP analysis and direct sequencing. These samples were obtained from healthy subjects without a family history of cancer (n = 10) and from patients with intestinal polyposis and/or early onset CRC (n = 16). The list of sequence variations identified in this control group is shown in Table 1. To validate the HRMA method, we subsequently tested 62 samples of unknown genotype from patients with clinical manifestations of MAP. Patients with one of the following characteristics were included in the initial and validation sets: 1. presence of ≥10 adenomatous or mixed adenomatous/hyperplastics polyps; 2. early-onset (< 40 years) sporadic CRC; 3. "multiple" polyps of unspecified number. The study was performed in accordance to the Helsinki Declaration (http://www.wma.net/en/30publications/10policies/b3/index.html). Informed consent was obtained from all patients for the use of specimens and clinical/pathological data for research purposes according to the guidelines established by the local ethical committee.

**Table 1:** *MUTYH *genotypes in the 26 samples used to set up the HRMA assay

AMPLICON	SSCP ANALYSIS	HRMA/SEQUENCING^1^	N. OF CASES
EXON 1	WT	WT	26
EXON 2	WT	WT	23
	WT	**c.[64G > A]+[=]**	1
	c.[64G > A]+[64G > A]	c.[64G > A]+[64G > A]	2
EXON 3	WT	WT	26
EXON 4	WT	WT	26
EXON 5	WT	WT	26
EXON 6	WT	WT	18
	c.[504+35G > A]+[=]	c.[504+35G > A]+[=]	7
	c.[481G > C]+[=]	c.[481G > C]+[=]	1
EXON 7	WT	WT	19
	c.[536A > G]+[536A > G]	c.[536A > G]+[536A > G]	2
	c.[536A > G]+[=]	c.[536A > G]+[=]	5
EXON 8	WT	WT	26
EXON 9	WT	WT	26
EXON 10	WT	WT	26
EXON 11	WT	WT	26
EXON 12	WT	WT	16
	c.[1014G > C]+[=]	c.[1014G > C]+[=]	6
	WT	**c.[1014G > C]+[1014G > C]**	1
	c.[1147delC]+[=]	c.[1147delC]+[=]	1
	c.[1023_1024insGA]**^2^**+[=]	c.[1023_1024insGA]**^2^**+[=]	1
	c.[1163T > C]+[=]	c.[1163T > C]+[=]	1
EXON 13	WT	WT	19
	c.[1187G > A]+[1187G > A]	c.[1187G > A]+[1187G > A]	1
	c.[1187G > A]+[=]	c.[1187G > A]+[=]	4
	c.[1227_1228dupGG]+[1227_1228dupGG]	c.[1227_1228dupGG]+[1227_1228dupGG]	1
	c.[1187-27C > T(+)1258C > A]**^3^**	c.[1187-27C > T(+)1258C > A]**^3^**	1
EXON 14	WT	WT	24
	c.[1437_1439delGGA]+[=]	c.[1437_1439delGGA]+[=]	1
	c.[1437_1439delGGA]+[1437_1439delGGA]	c.[1437_1439delGGA]+[1437_1439delGGA]	1
EXON 15	WT	WT	26
EXON 16	WT	WT	26

^**1**^In bold genetic variants identified by direct sequencing and HRMA, but not by SSCP.

^**2**^Novel variant identified in this study.

^**3**^These two variants were present in the same sample, but their phase is unknown.

WT = wild-type.

Genomic DNA was isolated from peripheral blood leukocytes by phenol/chloroform extraction. DNA concentrations were determined by Real-Time PCR on an ABI Prism 7000 Sequence Detection System (Applied Biosystems, Foster City, CA) with the Quantifiler^® ^Human Quantification Kit (Applied Biosystems, Foster City, CA).

### PCR amplification

The GenBank sequence NM_001128425.1 was used as the reference sequence for *MUTYH *cDNA. Primers for exons 3, 6 and 13 and for the 3' and 5' UTR regions were designed using Primer3 software (http://frodo.wi.mit.edu/primer3/), and their sequences are available upon request. All other exons and flanking intron sequences were amplified using previously reported oligonucleotides [22]. To increase sensitivity, we chose amplicon sizes not greater than 300 bp and we determined the folding characteristic of both primers and amplicons using the secondary structure profiling software DINAMelt (http://dinamelt.bioinfo.rpi.edu/hybrid2.php). The same primer sets were used for SSCP analysis, HRMA, and sequencing.

For each sample, PCR for HRMA analysis was performed under two different conditions: on the native sample, and on an 85:15 mixture of the test sample and a reference DNA sample homozygous for the wild-type sequence. Mixtures of reference and test samples allowed to obtain a condition of artificial heterozygosity [23,24].

PCR reactions for HRMA were carried out in a final volume of 15 μl containing 10-20 ng of template DNA, 0.05 U/μl of *Taq *Gold Polymerase (Applied Biosystems, Foster City, CA), 0.25 pmol/μl each primer, 1.5 μl of 10X reaction buffer, 0.9 μl of 25 mM MgCl_2_, 1.5 μl of 2.5 mM dNTPs and 0.15 μl of 50 μM intercalating dye Syto9^® ^(Invitrogen Corp., Carlsboard, CA). Cycling conditions were: 10 min at 95°C, followed by 45 cycles of 30 s at 95°C, 30 s at the annealing temperature, 30 s at 72°C, and a final extension at 72°C for 20 min.

PCR experiments for SSCP and sequencing analysis were carried out in a final volume of 20 μl and 30 μl, respectively, containing 100 ng of template DNA, 0.05 U/μl of *Taq *Polymerase (Applied Biosystems, Foster City, CA), 0.25 pmol/μl each primer, 1X reaction buffer, 1.5 mM MgCl_2_, 3 μl of 0.25 mM dNTPs. Cycling conditions were: 10 min at 95°C, followed by 40 cycles of 30 s at 95°C, 30 s at the annealing temperature, 30 s at 72°C, and a final extension at 72°C for 7 minutes.

### HRMA

All PCR products were electrophoresed in 1.2% agarose to verify the presence of a unique product of the expected size before performing HRMA.

To facilitate heteroduplex formation, PCR products not subjected to prior mixing with a wild-type sample were denatured at 95°C for 1 min and then rapidly cooled to 40°C for 1 min. Samples mixed with reference DNA were denatured as follows: 95°C for 8 min, 75°C for 6 min, 55°C for 6 min, 37°C for 6 min. HRMA was performed on a Rotor Gene™ 6000 Instrument (Corbett Research, Sidney, Australia). Fluorescence difference plots were generated for each amplicon as previously described [25]. Melting curve data for each reaction were acquired in a wide range of temperatures (70°C to 95°C), initially at a ramping rate of 0.1°C/sec, and subsequently at a ramping rate of 0.05°C/sec, to allow better resolution of heterozygous samples. Data were acquired and analyzed using software provided with the Rotor Gene™ 6000 Instrument (Corbett Research, Sydney, Australia). Wild-type controls were included in each experiment.

### DNA Sequencing

Direct sequencing of the PCR products was carried out using the Big Dye Terminator Cycle Sequencing kit (Applied Biosystems, Warrington, Cheshire, UK), according to manufacturer's instructions. Sequencing reactions were purified using the Dye Ex 2.0 Spin kit (Qiagen, Crawley, W. Sussex, UK) and samples were run onto an ABI 310 capillary sequencer (Applied Biosystems).

### SSCP analysis

SSCP analysis of the *MUTYH *coding sequence was performed on genomic DNA at 10°C and 20°C on 12.5% GeneGel Excel with a GenePhor apparatus (Amersham Biosciences, AB, Uppsala, Sweden). Silver nitrate was used to stain PCR products [26].

## Results

As a first step, we assessed the specificity and sensitivity of HRMA in a set of 26 samples whose genotypes had been preliminarily determined by SSCP and direct sequencing. All samples had been investigated by both SSCP and sequencing in order to compare the results obtained with the two methods. These allowed the detection of a total of 13 *MUTYH *point sequence variants in the 26 samples (Table 1). All variants were identified by SSCP, with the exception of the c.64G > A and c.1014G > C substitutions; these were detected in the heterozygous and homozygous state, respectively, only by sequencing. Six variants were present in multiple samples.

In order to verify whether HRMA allowed detection of homozygous sequence changes based on T_m _differences alone, the 26 samples were examined without prior mixing with the reference sample. In all samples harbouring *MUTYH *variants previously identified by SSCP, the shapes of the melting curves were altered compared to wild-type samples. All samples homozygous for *MUTYH *sequence changes recognized by SSCP analysis (c.64G > A, c.536A > G, c.1187G > A, c.1227_12289dupGG and c.1437_1439delGGA) could be reproducibly distinguished from the wild-type counterparts by a temperature shift in the melting curve. In addition, HRMA under these conditions allowed to identify one heterozygote for the c.64A allele, that had gone undetected by SSCP; this showed a melting curve clearly different from those of wild-type c.64G homozygotes (data not shown).

**Figure 1:**
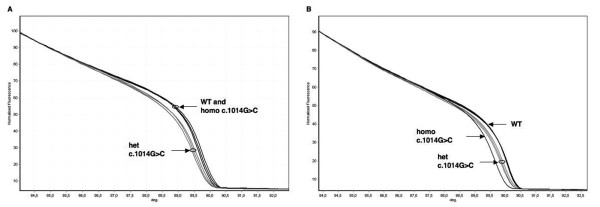
**HRMA of *MUTYH *exon 12**. The homozygous c.1014G > C transversion is visible with HRMA only when samples are mixed with wild-type DNA. A) HRMA of native test samples: the normalized melting curves of c.1014G > C heterozygotes are distinct from those of GG wild-type homozygotes; the melting profiles of GG and CC homozygotes are clustered together. B) After mixing with a reference DNA, the CC sample is clearly distinguishable from both heterozygous GC and homozygous GG samples. Homo: homozygote; het: heterozygote; WT: wild-type; deg: degrees (°C).

We then re-examined all samples after mixing with a reference genomic DNA. All genotypes reported in Table 1, including the homozygote for the c.1014G > C (p.Gln388His) variant (Figure 1), were detected by HRMA under these conditions. Melting curves were reproducible in duplicates of each sample and among different samples carrying the same mutations.

**Figure 2:**
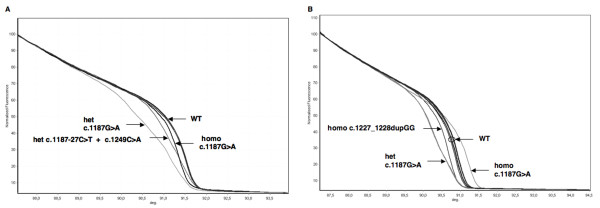
**HRMA of *MUTYH *exon 13**. All heterozygous and homozygous variants in exon 13 are clearly distinguishable from each other and from wild-type samples without mixing with reference DNA. A) Normalized melting curves obtained for samples carrying variants c.1187G > A (both homozygous and heterozygous), c.1187-27C > T + c.1249C > A (double heterozygous), and wild-type samples; B) Normalized melting curves obtained for c.1187G > A (both homozygous and heterozygous) and homozygous c.1227_1228dupGG, and wild-type samples. Homo: homozygote; het: heterozygote; WT: wild-type; deg: degrees (°C).

**Figure 3:**
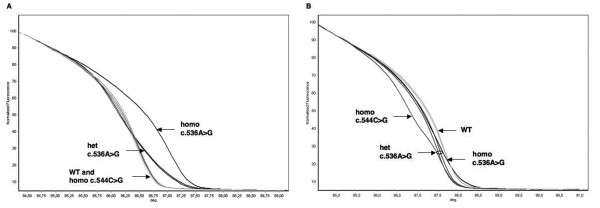
**HRMA of *MUTYH *exon 7**. Two mutations (c.536A > G and c.544C > G) were identified in this exon. A) Normalized melting curves of c.536A > G heterozygotes and homozygotes are clearly distinct from wild-type homozygotes; on the other hand, wild-type and c.544C > G homozygous samples have identical melting profiles; B) After mixing with a reference DNA, the c.544C > G homozygous sample is clearly differentiated from wild-type, c.536A > G heterozygous and homozygus samples. Homo: homozygote; het: heterozygote; WT: wild-type; deg: degrees (°C).

The two most frequent Caucasian mutations (c.536A > G and c.1187G > A) were clearly detected both in the heterozygous and homozygous state and were associated with significant curve shape changes (Figures 2, 3). In addition to the c.1187G > A mutation, other sequence variations in exon 13 were recognized (Figure 2). These included one homozygous insertion (c.1227_1228dupGG), one heterozygous intronic C > T transition (c.1187-27C > T), and one heterozygous C > A transversion (c.1258C > A). The associated curves could be clearly distinguished from those of wild-type and c.1187G > A samples. The c.1187-27C > T and c.1258C > A substitutions were present in the same sample.

Abnormal melting profiles were clearly identified also for the remaining 5 variants. Moreover, reproducible melting profiles were obtained for samples carrying the polymorphic variants (c.64G > A, c.504+35G > A, c.1014G > C) located in exons 2, 6 and 12.

The mutation detection sensitivity was 100% compared to direct sequencing in samples subjected to mixing with a reference DNA. Specificity was 100%, since all abnormal profiles were associated with sequence changes. Sensitivity of HRMA performed without mixing with a reference DNA was 94% (17/18 *MUTYH *variant genotypes recognized).

We then performed a blinded analysis by HRMA in a set of 62 further samples of unknown genotype, obtained from patients with clinical characteristics suggestive of MAP. Again, the experiments were performed both with and without mixing samples with the reference DNA. All samples were also investigated by direct sequencing. We identified 15 different sequence variations in this group: 8 missense, 5 intronic and 2 in the 5'UTR (Table 2). All variations were identified both in mixed and unmixed samples, with the exception of the homozygous c.1014G > C polymorphic variant and of a base substitution in exon 7 (c.544C > G; p.Arg182Gly); the latter was present in a single sample, in homozygosity, and could be detected only after mixing with reference DNA (Figure 3).

**Table 2:** *MUTYH *sequence variants identified by HRMA in a set of 62 samples of unknown genotype

AMPLICON	GENOTYPES IDENTIFIED^1^	N. OF CASES
5'UTR	WT	60
	c.[-127C > T]+[=]	1
	**c.[-205C > A]+[=]**	1
EXON 2	WT	54
	c.[64G > A]+[=]	4
	c.[64G > A]+[64G > A]	1
	**c.[37G > A]+[=]**	1
	c.[56G > A]+[=]	1
	c.[157+30A > G]+[=]	1
EXON 6	WT	55
	c.[504+35G > A]+[=]	7
EXON 7	WT	58
	c.[536A > G]+[=]	2
	**c.[544C > G]+[544C > G]**	1
	c.[1187-27C > T]+[=]	1
EXON 8	WT	61
	c.[690+21C > A]+[=]	1
EXON 12	WT	38
	c.[1014G > C]+[=]	19
	c.[1014G > C]+[1014G > C]	5
EXON 13	WT	7
	c.[1187G > A]+[=]	1
EXON 15	WT	61
	c.[1477-40G > C]+[=]	1
EXON 16	WT	58
	c.[1544C > T]+[=]	4

^**1**^In bold novel variants identified in this study by HRMA. WT = wild-type.

**Figure 4:**
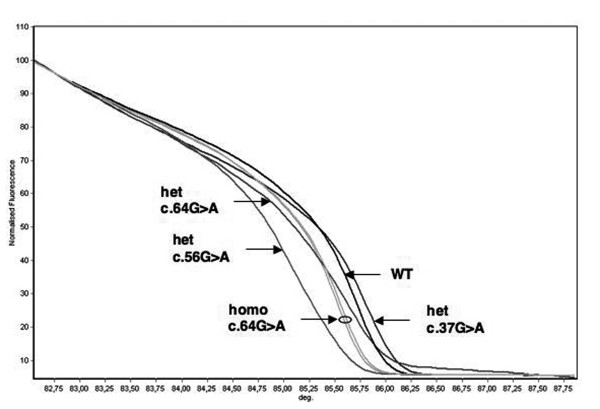
**HRMA of *MUTYH *exon 2**. Four genotype combinations, in addition to wild-type, are distinguishable for this exon based on melting profile analysis, without prior mixing with a reference DNA. Homo: homozygote; het: heterozygote; WT: wild-type; deg: degrees (°C).

Four different variants were identified in exon 2: the intronic polymorphism c.157+30A > G, the polymorphic missense substitution (c.64G > A; p.Val22Met), also present in the first sample series investigated, and two additional heterozygous base substitutions leading to aminoacid changes, c.37G > A (p.Ala13Thr) and c.56G > A (p.Arg19Gln). The melting profiles associated with the latter two rare variants could clearly be distinguished from those of samples homozygous and heterozygous for p.Val22Met (Figure 4).

All samples were also subjected to sequencing, and no further alterations were observed. Again, the sensitivity and specificity of mutation detection by HRMA analysis in this blinded set of samples was 100% in experiments performed on mixtures of test and reference DNAs, while it was lower (88%; 15/17 variant genotypes detected) in unmixed samples.

HRMA also allowed to identify three polymorphic variants in the heterozygous state, located in intron 8 (c.690+21C > A), intron 14 (c.1477-40G > C) and exon 16 (c.1544C > T; p.Ser515Phe), respectively. These were observed both with and without mixing the samples with the reference DNA (data not shown).

To the best of our knowledge, four of the sequence variants identified in this study (c.-205C > A, c.37G > A, c.544C > G, and c.1023_1024insGA) have not previously been reported in the literature nor are they included in the *MUTYH *Leiden Open Variation Database (LOVD; http://chromium.liacs.nl/LOVD2/colon_cancer/home.php) database.

## Discussion

We have shown that *MUTYH *mutations can be identified with high sensitivity by HRMA both in the heterozygous and in the homozygous state. HRMA sensitivity was higher compared to SSCP and equal to direct sequencing, the gold standard for assessing sequence variations. We have analyzed 26 samples containing 13 DNA sequence variations located in 6 exons, using both HRMA and SSCP. Sensitivity of HRMA analysis appeared superior to that of SSCP, as previously reported [27]. In particular, HRMA allowed the detection of heterozygous and homozygous sequence changes (c.64G > A and c.1014G > C) that had not been identified by SSCP.

HRMA has been introduced very recently in molecular genetic diagnostic laboratories and has shown several advantages: cost-effectiveness, simplicity, absence of post-PCR treatment, and, particularly, the swiftness to identify genetic variants or mutations. So far, this technique has been mainly used for the detection of mutations in genes involved in X-linked and autosomal dominant diseases, such as *OTC, BRCA1, BRCA2 *and *TP53 *[15,28,29]. Applications to autosomal recessive disorders have been more limited, as homozygous mutations, especially G > C and A > T substitutions and small insertions/deletions (e.g., p.Phe508del in *CFTR*) may not be associated with melting curve changes and consequently may escape detection [30]. Nevertheless, the technique has also been successfully applied to investigate genes associated with autosomal recessive conditions, such as *CFTR, PEX6, ABCA4, ATP7B, C2ORF71 *[18,19,31-33]. It has also been shown that the sensitivity limits of HRMA for the detection of homozygous sequence changes can be overcome by mixing the test DNA sample with a reference wild-type DNA [16-18,28,29,34].

López-Villar et al. [20] have recently used HRMA to detect heterozygous and homozygous variants in *MUTYH *exons 7, 12 and 13 in a series of 82 patients with a phenotype suggestive of MAP. The experiments were set up without mixing samples with a reference DNA. Hence, homozygotes for common variants in these exons, such as c.1014G > C, as well as for rare nucleotide changes, could have gone undetected, since heteroduplexes could not be formed. In addition, the melting profiles of samples heterozygous for the common c.1187G > A and for the rare c.1276C > T (p.Arg426Cys) mutation appeared to be overlapping.

Partial *MUTYH *mutation screening by HRMA limited to exons 7, 13 and 15, has been recently performed in a series of cutaneous melanomas [21]; however, also in this latter study, the samples had not been premixed with reference DNA. Therefore, so far HRMA-based approaches for the detection of *MUTYH *mutations have been limited to few targeted regions of the gene. In addition, the sensitivity of HRMA compared to that of other mutation screening methods was not assessed in either study.

Although the majority of *MUTYH *mutations present in Caucasian MAP patients are located in exons 7 and 13, several additional pathogenic variants are scattered over all other exons of the gene in the Caucasian population [35]. In addition, the distribution of *MUTYH *mutations differs among populations [36]. Therefore, in order to allow maximum sensitivity, mutation scanning needs to be performed on all *MUTYH *exons.

A similar situation applies to the *CFTR *gene, where population-specific mutations are observed in different populations. For this gene, it has been demonstrated that identification of all point mutations, both at the heterozygous and homozygous state, can be achieved by HRMA when the whole coding sequence is investigated [18]. In addition, it has been observed that homozygotes for the common Caucasian p.Phe508del mutation can only be identified when the formation of heteroduplexes is induced by mixing with wild-type DNA [18].

We therefore developed an improved HRMA-based *MUTYH *analysis protocol that scans the entire coding region and the UTRs and increases sensitivity by mixing the DNA test with a reference DNA. Our results confirm that the common Caucasian mutations in the *MUTYH *gene can be reliably identified by HRMA even when present in the homozygous state. We also found that the T_m_s of samples homozygous for either of the two common *MUTYH *mutations are consistently different from those of their wild-type counterparts; this allows simple and rapid detection of homozygotes for these frequent variations in native test DNA samples, without need to add reference wild-type DNA.

In addition, complete HRMA scanning of *MUTYH *clearly differentiated all samples containing DNA sequence variations, including small insertions and deletions, from wild-type DNA. Importantly, samples heterozygous for different variants located within the same amplicon displayed distinct melting profiles. However, it has been pointed out that occasionally the melting curves of samples containing two linked variants located in the same amplicon may be undistinguishable from those observed in simple heterozygotes [37]. Hence, we advocate that the mixing strategy should be used also for the purpose of identifying all possible heterozygote combinations.

The availability of a rapid and inexpensive method for the detection of *MUTYH *mutations is important for the identification of individuals at increased CRC risk. Since the phenotype associated with *MUTYH *mutations is highly variable, the diagnosis of MAP is difficult on clinical grounds alone [5-7,38,39]. In addition, several studies have shown that monoallelic *MUTYH *mutation carriers may have a moderately increased risk of CRC compared to the general population [40-43]. Since the frequency of simple heterozygotes for *MUTYH *mutations may be as high as 0.02 in Caucasian populations [44], *MUTYH *testing could become a widespread screening test to identify individuals at risk for CRC in the general population.

## Conclusions

We have set up a very fast, simple and non-expensive mutation scanning method that identifies the most common *MUTYH *mutations and known polymorphic variants, as well as rare DNA sequence changes spread in the coding sequence and 5' UTR. In Caucasian patients, molecular screening by HRMA can be performed in two steps: the first one to identify common variants in exons 7 and 13 without mixing with a reference DNA; the second one should be performed using a mixture of test and control DNA, to detect more rare changes in the whole coding sequence when no or a single mutation has been identified in the first stage. The implementation of a simple and sensitive method for *MUTYH *mutation scanning is relevant for the diagnosis of CRC susceptibility and for the identification of at risk individuals, who should be advised intensive surveillance.

## Declaration of competing interests

The authors declare they have no competing interests.

## Authors' contributions

RT designed the study, performed the experiments and wrote the manuscript; AA contributed to carry out the experiments; FC, CO and RS were involved in the design of HRMA experiments and critically read the manuscript; FT and RV were involved in the selection of patients and in the provision of biological samples, MG conceived the study, participated in its design and helped to draft the manuscript. All authors have read and approved the final manuscript.

## Pre-publication history

The pre-publication history for this paper can be accessed here:

http://www.biomedcentral.com/1471-2407/11/305/prepub
